# Circular RNA* circPPM1F* modulates M1 macrophage activation and pancreatic islet inflammation in type 1 diabetes mellitus

**DOI:** 10.7150/thno.48264

**Published:** 2020-08-29

**Authors:** Caiyan Zhang, Xiao Han, Lan Yang, Jinrong Fu, Chengjun Sun, Saihua Huang, Wenfeng Xiao, Yajing Gao, Qiuyan Liang, Xiang Wang, Feihong Luo, Wei Lu, Yufeng Zhou

**Affiliations:** 1 Institute of Pediatrics, Children's Hospital of Fudan University, and the Shanghai Key Laboratory of Medical Epigenetics, International Co-laboratory of Medical Epigenetics and Metabolism, Ministry of Science and Technology, Institutes of Biomedical Sciences, Fudan University, Shanghai 200032, China.; 2 National Health Commission (NHC) Key Laboratory of Neonatal Diseases, Fudan University, Shanghai, China.; 3 Department of Pediatric Endocrinology and Inherited Metabolic Diseases, Children's Hospital of Fudan University, Shanghai, 201102, China.

**Keywords:** Circular RNA, Type 1 diabetes mellitus, Macrophage activation, RNA-binding protein, Islet injury

## Abstract

**Rationale:** Macrophages play critical roles in the pathogenesis of type 1 diabetes mellitus (T1DM). Circular RNAs (circRNAs) are a novel class of endogenous RNAs with covalently closed loop structures, implicated in various disease processes. However, their impact on macrophage activation and T1DM pathogenesis remains elusive.

**Methods:** circRNA expression profiles of peripheral blood mononuclear cells (PBMCs) from T1DM children were determined by whole transcriptome microarray. Bioinformatics, quantitative real-time PCR, Western blot, RNA immunoprecipitation (RIP), cell co-culture, cell proliferation, and cell apoptosis assays were performed to investigate the expression, function, and regulatory mechanisms of *circPPM1F in vitro*. The regulatory role of *circPPM1F in vivo* was evaluated in the streptozocin-induced diabetic mouse model.

**Results:** We identified 27 upregulated and 31 downregulated differentially expressed circRNAs in T1DM patients. *circPPM1F*, a circRNA with unknown function, was dominantly expressed in monocytes and significantly upregulated in T1DM patients. Functionally, *circPPM1F* promoted lipopolysaccharide (LPS)-induced M1 macrophage activation via enhancement of the NF-κB signaling pathway. Mechanistically, *circPPM1F* competitively interacted with HuR to impair the translation of protein phosphatase, Mg^2+^/Mn^2+^ dependent 1F (PPM1F), thus alleviating the inhibitory effect of PPM1F on the NF-κB pathway. Moreover, eukaryotic initiation factor 4A-III (EIF4A3) and fused in sarcoma (FUS) coordinately regulated *circPPM1F* expression during M1 macrophage activation. In addition, *circPPM1F* could exacerbate pancreas injury in the streptozocin-induced diabetic mice by activation of M1 macrophages *in vivo*.

**Conclusions:** *circPPM1F* is a novel positive regulator of M1 macrophage activation through the *circPPM1F*-HuR-PPM1F-NF-κB axis. Overexpression of *circPPM1F* could promote pancreatic islet injury by enhancing M1 macrophage activation and *circPPM1F* may serve as a novel potential therapeutic target for T1DM in children.

## Introduction

Type 1 diabetes mellitus (T1DM, also known as insulin-dependent diabetes) is a chronic autoimmune disease, driven by the interplay between individual genetics and environmental triggers 1, 2. T1DM is diagnosed at all ages 3, 4, and is characterized by aberrant autoimmune destruction of pancreatic islet β cells, resulting in a complete lack of insulin synthesis and necessitating lifelong hormone replacement therapy 5. Recent studies have revealed that a spike in the incidence of T1DM was found at ages 10-14 years, and worldwide estimates of numbers of children and adolescents with T1DM continue to increase 6.

Emerging evidence has suggested that, throughout the immune system, macrophages play a critical role in insulitis, and support autoimmune T cells to aggravate the infiltration of inflammatory cells during T1DM 7. Macrophages have been classified as two extreme examples on the activation spectrum: classically activated macrophages (M1) with a pro-inflammatory phenotype and alternatively activated macrophages (M2) with an anti-inflammatory phenotype. Generally, lipopolysaccharide (LPS) and interferon (IFN)-γ are the main stimulating factors for M1 macrophage activation, while interleukin (IL)-4 and IL-13 can activate macrophages to M2 8, 9. Notably, M1 macrophage activation drives pathogenesis and progression of diabetes through exacerbation of inflammatory responses via secretion of inflammatory cytokines 10, 11. For example, Arnush et al. found M1 macrophages promoted destruction of β cells in T1DM mice through excessive production of IL-1β 12. In addition, Tim-3 exacerbated podocyte injury via M1 macrophage activation in streptozocin (STZ)-induced diabetic nephropathy 13. Importantly, blocking macrophage infiltration into the pancreas or restraining macrophage activation in diabetic mice maintained pancreas function and prevented T1DM initiation 14, 15. Therefore, elucidating the underlying mechanism of M1 macrophage activation is likely to lead to a better understanding of the pathogenesis and therapy of T1DM.

Circular RNA (circRNA) is a covalently closed loop molecule ligated by a 3′-5′ phosphodiester bond at the junction site, which is generated through back-splicing and often considered to be a byproduct of aberrant splicing 16, 17. Recently, high-throughput sequencing has been used to identify thousands of endogenous circRNA species in mammalian cells, which are stable, conserved, and abundant 18, 19. By modulation of gene expression, interaction with RNA binding proteins (RBPs), circRNAs directly or indirectly participate in autoimmune diseases and cancers. For instance, *circ-RasGEF1B* promotes the antigen presentation process by positively regulating the stability of intercellular adhesion molecule 1 (*ICAM-1*) mRNA in macrophages exposed to LPS 20. Additionally, *circZKSCAN1* negatively regulates cancer stem cells by physically binding fragile X mental retardation protein (FMRP) against cell cycle and apoptosis regulator 1 (CCAR1) complex in hepatocellular carcinoma 21. However, the roles of circRNAs in regulating M1 macrophage activation and T1DM are currently undefined.

In this study, we conducted a genome-wide analysis of circRNA expression profiles in peripheral blood mononuclear cells (PBMCs) from T1DM patients and healthy controls and identified a novel circRNA, *circPPM1F*, which was significantly upregulated in the PBMCs of T1DM patients. Functionally, *circPPM1F* could promote LPS-induced M1 macrophage activation via enhancement of NF-κB signaling. Mechanistically, *circPPM1F* competitively interacted with HuR to impair the translation of PPM1F, thus alleviating the inhibitory effect of PPM1F on the NF-κB pathway. Moreover, we found that EIF4A3 and FUS participated in the maintenance of high levels of *circPPM1F* expression. In addition, *circPPM1F* could exacerbate pancreas injury in STZ-induced diabetic mice by activating M1 macrophages. Overall, our study indicates that *circPPM1F* plays an important role in the development of T1DM and suggests a potential therapeutic target for T1DM.

## Materials and Methods

### Human study subjects

T1DM patients were diagnosed according to the criteria of American Diabetes Association (ADA) 22. Human peripheral blood samples were collected from T1DM patients (8.5 ± 0.6 y) and age-matched healthy controls (8.0 ± 1.2 y), following informed consent from all patients, at the Children′s Hospital of Fudan University, Shanghai, China. Detailed characteristics of the study subjects are presented in Table S1. The study was approved by the Research Ethics Board of the Children′s Hospital of Fudan University [No. (2016) 96].

PBMCs were isolated using Ficoll-Hypaque (GE Healthcare, USA). Briefly, peripheral blood was mixed with phosphate-buffered saline (PBS) and overlaid on the Ficoll-Hypaque solution (density: 1.077 g/mL). Following centrifugation at 400 ×g for 30 min, PBMCs were aspirated from the interface. The cell pellet was washed twice with PBS and resuspended in TRIzol for subsequent RNA extraction and quantitative real-time PCR (qRT-PCR). In addition, CD14^+^, CD3^+^, and CD19^+^ cells were sorted from PBMCs with a magnetic cell sorting system (Miltenyi Biotec, Germany). Sorted cells were subjected to RNA extraction and qRT-PCR.

### RNA extraction and quantitative real-time PCR

The nuclear and cytoplasmic fractions or total RNA were extracted using TRIzol (Invitrogen, USA) followed by reverse transcription of mRNAs and circRNAs using PrimeScript II 1st Strand cDNA Synthesis Kit (Takara, Japan) according to the standard manufacturer′s instructions. A qRT-PCR assay was performed to measure mRNAs and circRNAs expression with SYBR^®^ Premix Ex Taq™ II (Takara, Japan) using the Roche 480 Real Time PCR System. β-actin served as internal control for mRNAs and circRNAs. Relative quantification (2^-ΔΔCT^) was used for results analysis. The primers sequences in qRT-PCR are listed in Table S2.

### Microarray

The circRNA microarray analysis was performed at the Shanghai Biotechnology Corporation. In brief, Total RNA of PBMCs from T1DM patients (n = 4) and age-matched healthy controls (n = 4) were extracted and purified followed by amplification and labeling with a Low Input Quick Amp WT Labeling Kit. Labeled cRNAs were purified using the RNeasy mini kit. Each slide was hybridized with 1.65 μg Cy3-labeled cRNA for 17 h using a gene expression hybridization kit, and then scanned with an Agilent microarray scanner using default settings. Data were extracted with the Feature Extraction software 10.7 (Agilent Technologies, Santa Clara, CA, US). Raw data were normalized by the Quantile algorithm and limma package in R. Overall, 88750 circRNAs were tested.

### Cell culture and transfection

The human THP1 cell line was obtained from the American Type Culture Collection (ATCC, Manassas, VA, USA), and MIN6 and Raw264.7 cells were obtained from the Fudan IBS cell resource center (FDCC, Shanghai, China). THP1 and MIN6 cells were maintained in RPMI-1640 medium (Gibco, Gaithersburg, MD, USA) with 10% fetal bovine serum (FBS, Gibco) and 1% penicillin/streptomycin (Gibco), and the MIN6 cell culture medium was supplemented with 50 μM 2-mercaptoethanol. Raw264.7 cells were maintained in Dulbecco′s modified Eagle′s medium with high glucose (DMEM, Gibco) supplemented with 10% FBS. All cells were kept in a humidified cell incubator with 5% CO_2_ at 37 °C.

Cells were transiently transfected with Lipofectamine RNAiMAX reagent (Invitrogen, USA) and chemically synthesized si-*circPPM1F*, siPPM1F, siHuR, siEIF4A3, or siFUS (GenePharma, Shanghai, China) according to standard protocols. Cells were transiently transfected with 500 ng/mL ectopic expression vector of *circPPM1F* with Lipofectamine 2000 reagent (Invitrogen, USA), according to the manufacturer's instructions. qRT-PCR assay was used to assess RNA expression levels 48 h after transfection. Relative sequences of siRNA are listed in Table S3.

For the activation of THP1-derived macrophage, THP1 cells were treated as previously described 23. Briefly, THP1 were treated with 500 ng/mL phorbol 12-myristate 13-acetate (PMA) for 48 h to induce the differentiation of THP1 into macrophages (THP1 macrophages), and then 200 ng/mL LPS was used to induce M1 macrophage activation.

### Western blot

Total protein was extracted using Radio Immunoprecipitation Assay (RIPA) lysis buffer (Thermo Scientific, USA). Lysates were resolved by electrophoresis, transferred to polyvinylidene fluoride (PVDF) membranes, and probed with antibodies directed against PPM1F (Abcam); HuR, phosphorylated NF-κB p65 (Ser536) (p-p65), p65, phosphorylated p38 (T180/Y182) (p-p38), p38, phosphorylated JNK (T183/Y185) (p-JNK), JNK, phosphorylated ERK1/2 (T202/Y204) (p-ERK1/2), ERK1/2, phosphorylated m-TOR (Ser2448) (p-mTOR), mTOR, phosphorylated Stat3 (Y705) (p-Stat3), Stat3, Bcl2 (Cell Signaling Technology, USA); Bax (EMD Millipore, USA), and β-tubulin (Abcam, USA). The bands were detected by developing with chemiluminescent HRP substrate (Thermo Scientific, USA), and intensity of bands was determined by imaging with a Molecular Imager^®^ (Bio-RAD, ChemiDocTM XRS+ Imaging System, USA). All results were normalized to those of β-tubulin, which was used as a loading control. Detailed characteristics of the antibodies are presented in Table S4.

### RNase R digestion

Four micrograms total RNA from THP1 cells was either untreated (control) or treated with 20 units of RNase R (Epicenter; USA, RNR07250) in the presence of 1 × Reaction Buffer, and incubated for 30 min at 37 °C. The digested RNA was isolated using acid phenol-chloroform (5:1). Then reverse transcription and qRT-PCR were performed, as described in the RNA extraction and qRT-PCR section.

### RNA stability

THP1 cells (1 × 10^5^) were placed in 24-well plates and treated with 250 ng/mL actinomycin D (Act D, Sigma) added to the cell culture medium. The levels of *circPPM1F* and *PPM1F* were detected at 0, 3, 6, 12, and 24 h.

### Subcellular fractionation and localization

Nuclear and cytosolic fractions were separated using the Nuclear and Cytoplasmic Extraction Kit (Cwbio, China). A total of 1 × 10^7^ cells were harvested, re-suspended in 1 mL of Nc-buffer A and 55 µL Nc-Buffer B, and incubated for 20 min on ice. Cells were then centrifuged for 15 min at 12, 000 ×g; the resulting supernatants (containing the cytoplasmic component) and nuclear pellets were used for RNA extraction.

### RNA immunoprecipitation

The RNA immunoprecipitation (RIP) assay was performed using the Magna RIP RNA-Binding Protein Immunoprecipitation Kit (EMD Millipore Corp., Billerica MA, USA). Two 10-cm culture dishes of THP1 macrophages (1 × 10^7^/dish) were harvested, centrifuged and re-suspended using 100 μL RIP lysis buffer supplemented with protease and RNase inhibitors, and 5 μg IgG or HuR antibody-coated beads were incubated with the cell lysates under rotary agitation at 4 °C overnight. Following proteinase K treatment, the immunoprecipitated RNAs were extracted and reversely transcribed as described in the RNA extraction and qRT-PCR section. Levels of *circPPM1F* and *PPM1F* were detected by qRT-PCR assay.

### *circPPM1F* vector construction

3D5 is a modified plasmid based on the pZW1 vector. Two reverse complementary sequences helped to form a circular structure derived from the *POLR2A* gene and were inserted upstream of the restriction site *Xho* I and downstream of *Pac* I. Primer 1 or primer 2 (Table S2) was used to amplify the sequence of *circPPM1F* upstream (~340 bp) or downstream (~3800 bp) of cDNA or gDNA. Two PCR segments were inserted into the 3D5 vector following digestion with restriction enzymes *Xho* I and *Pac* I using a seamless cloning assay (Figure S1), and the product was transformed into STBL3 competent cells. The recombinant vector sequences were validated by Sanger sequencing.

### Conditional media collection and cell treatment

Raw264.7 cells were seeded in six-well plates, and transfected with 3D5-*circPPM1F*, or corresponding negative control plasmids according to the cell transfection method used. Next, 48 h post transfection, the transfected cells were stimulated with 200 ng/mL LPS for 20 h. Culture media were gathered and centrifuged at 3000 rpm at 4 °C for 30 min, after which the supernatants (conditional media) were collected. The conditional media were used for treatment of MIN6 cells, at a ratio of 3:1 with complete medium.

### Enzyme-linked immunosorbent assay (ELISA)

The protein levels* of IL-6* and* TNF-α* in the conditional media were measured with the mouse *IL-6* and* TNF-α* DuoSet ELISA kit (eBioscience) according to the manufacturer's instructions. A microplate reader (Synergy4; BioTek, Winooski, VT, USA) was used to read the absorbance at 450/570 nm.

### Cell proliferation assay

The CCK8 assay was performed to assess the proliferative ability of MIN6 cells according to the manufacturer's instructions (DOJINDO Molecular Technologies, Inc., Kumamoto, Japan). Briefly, MIN6 cells (1.8 × 10^3^) were placed in 96-well plates and cultured in conditional medium. Each sample was assayed in triplicate. Cell viability was determined at 0, 24, 48, and 72 h using 10 μL CCK8 solution treatment for 2 h. The optical density of each well was assessed using a Microplate reader (Synergy4; BioTek, Winooski, VT, USA) at 450 nm.

### Cell apoptosis assay

MIN6 cells were cultured in six-well plates (1 × 10^5^ cells/well) for 48 h using conditional media, followed by H_2_O_2_ (500 μΜ in FBS-free medium) treatment for 15 h, and then pooled together after trypsin without EDTA digestion. Cell apoptosis was analyzed using the Annexin V-FITC/Propidium Iodide (PI) Apoptosis Detection Kit (BD Pharmingen, New York, USA, #556547) according to the manufacturer's instructions. MIN6 cells were stained with FITC and PI and then analyzed by fluorescence-activated cell sorting using FACS Canto II (BD Biosciences, San Jose, CA, USA). The cell apoptosis data were analyzed by FlowJo V10 software (Tree Star, San Francisco, CA, USA). Cells of quadrant 4 that were considered viable were FITC Annexin V and PI negative; cells of quadrant 3 that were in early apoptosis were FITC Annexin V positive and PI negative; cells of quadrant 2 that were in late apoptosis were both FITC Annexin V and PI positive; and cells of quadrant 1 that were necrotic were FITC Annexin V negative and PI positive. The percentages of cell apoptosis were the sum of that from early apoptosis and late apoptosis.

### Animal studies in the STZ-induced diabetic mouse model

C57BL/6 mice (8-week-old, male) were purchased from Shanghai Slac Laboratory Animal Co. Ltd. All mice were housed at the Experiment Animal Center of Children′s Hospital of Fudan University at room temperature 22 °C under a 12:12 h light/dark cycle, and were provided with rodent chow and tap water. The mice were randomly divided into four experimental groups: Control, STZ, STZ+pZW1, and STZ+circPPM1F group. On day 0, STZ+circPPM1F and STZ+pZW1 group mice were intraperitoneally (*i.p.*) injected with *circPPM1F* plasmid (8 μg/mouse, 150 μL) and an equal amount of pZW1 using the Entranster™-*in vivo* Transfection Reagent (Engreen, China), respectively; control and STZ group mice were injected with PBS. From day one, STZ, STZ+pZW1, and STZ+circPPM1F group mice were *i.p.* injected with repeated low doses of STZ (50 mg/kg body weight/day, 300 μL/mouse for five consecutive days); control group mice were injected with sodium citrate buffer (Fankew, FK4006). On day seven, mice were injected *circPPM1F*, control plasmids, or PBS again, and then received two injections per week. The animal experiments were repeated independently 3 times.

Body weight was measured weekly from day one. Blood glucose was also detected weekly from the tail vein blood using a Roche glucose reader (Roche Diagnostics GmbH, Germany). All mice were fasted for 12 h before glucose detection. On day 29, mice were sacrificed and pancreas tissues dissected and analyzed for pathology.

This project complied with the institutional guidelines and laws for the care and use of laboratory animals. The study was approved and overseen by the Animal Studies Committee of the Children′s Hospital of Fudan University [No. (2016) 96].

### Immunohistochemistry and Immunofluorescence studies

Pancreas tissues were fixed with 4% paraformaldehyde and then embedded in paraffin and cut into slices. For immunohistochemical analysis, a portion of paraffin sections were routinely stained with hematoxylin and eosin (H&E), while others were incubated with antibodies against Ki-67 (Signalway Antibody, USA), Insulin (Proteintech, USA), and F4/80 (Cell Signaling Technology, USA). Islets from consecutive tissue cross-sections were photographed at identical exposure conditions and magnification (400×) using a microscope (Leica). For immunofluorescence analysis, paraffin slides of 3-4 μm were prepared, and then incubated with rabbit anti-mouse F4/80 and rabbit anti-mouse iNOS (Servicebio, China). FITC (green) and Cy3 (red)-conjugated goat anti-rabbit IgG (Servicebio) were used to visualize F4/80 and iNOS, respectively. DAPI (4′, 6-diamidino-2-phenylindole) was used to stain the cell nuclei (blue). Images were captured with a fluorescence microscope (Leica). Detailed characteristics of the antibodies used are presented in Table S4.

### Preparation of pancreas single cell suspensions and flow cytometry

Pancreas tissues were collected into the gentleMACS C Tubes (#130-093-237; Miltenyi Biotec, Germany) containing the enzyme mix of the Multi Tissue Dissociation Kit 1 (#130-110-201, Miltenyi Biotec,Germany) in serum-free RPM-1640 and cut into 2 × 4-mm pieces. Tissues were subjected to the run program Multi_37C_m (30 min) on the gentleMACS Octo Dissociator with Heaters (#130-096-427, Miltenyi Biotec, Germany). The cell suspensions were passed through a 70-μm MACS SmartStrainer (#130-098-462, Miltenyi Biotec, Germany), followed by centrifuging at 300 ×g for 7 min at 4 °C. All isolated cells were suspended in 100 μL iced PBS supplemented with 2% FBS. Cells were then counted with trypan blue and processed for flow cytometry in as indicated below.

For surface marker analysis, cells were stained with anti-mouse F4/80 (eBioscience, USA) for 30 min at 4 °C. For intracellular cytokine staining, cells were fixed and permeabilized and labeled with anti-mouse iNOS (eBioscience, USA) after anti-F4/80 staining. The concentration of each antibody was used according to the recommended product protocol. Cells were examined by flow cytometry using a BD FACSCanto II instrument (BD Biosciences, San Jose, CA, USA) and analyzed with FlowJo V10 software (Tree Star, San Francisco, CA, USA). Detailed characteristics of the antibodies used are presented in Table S4.

### Statistical analysis

Results from three independent experiments are expressed as mean ± standard error of the mean (SEM). The two-tailed Student's *t*-tests were used for comparisons between two groups, and one-way analysis of variance (ANOVA) was used for multifactorial comparisons. A *p*-value of < 0.05 was considered statistically significant. The relationship between *circPPM1F* and *IL-6*, *IL-1β, TNF-α, EIF4A3* or* FUS* was tested using Pearson's correlation and linear regression. Statistical analyses were performed with SPSS v.19.0 software or GraphPad Prism 7.0 software.

## Results

### *circPPM1F* was upregulated in PBMCs from children with type 1 diabetes mellitus

**Figure 1:**
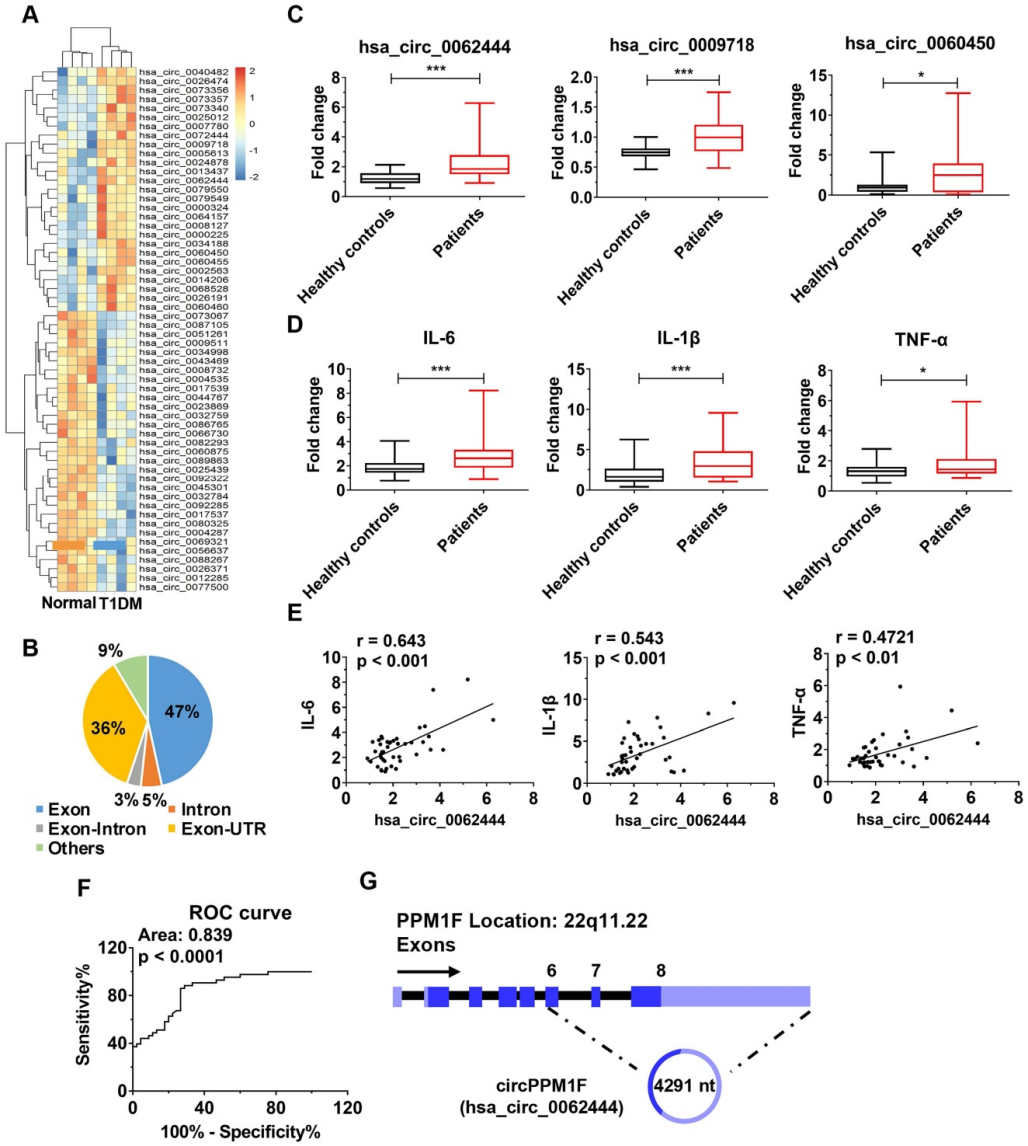
***circPPM1F* is upregulated and is associated with inflammatory cytokines in peripheral blood mononuclear cells from type 1 diabetes mellitus patients. A.** Heatmap showing 27 upregulated and 31 downregulated differentially expressed circRNAs in peripheral blood mononuclear cell (PBMC)s of type 1 diabetes mellitus (T1DM) patients (n = 4) and age-matched healthy controls (n = 4) (fold change > 2.0, *p* < 0.05). **B.** Composition of the circRNAs according to the position of the gene in the transcript. **C, D.** Analyses of the expression levels of *hsa_circ_0062444, hsa_circ_0009718, hsa_circ_0060450, IL-6, IL-1β,* and* TNF-α* (normalized to β-actin) in PBMCs from 43 T1DM patients and 45 healthy controls, as determined by qRT-PCR. **E.** Correlation analysis of the expression of *hsa_circ_0062444* and *IL-6, IL-1β* or* TNF-α* in 43 T1DM patients (Pearson's correlation). **F.** Receiver operating curve (ROC) analysis of *circPPM1F* levels in the study population. **G.** Schematic illustration showing the location of *hsa_circ_0062444* in host gene *PPM1F*. Data are presented as mean ± SEM. *p ≤ 0.05, ***p ≤ 0.001.

To identify differentially expressed circRNAs in T1DM, we first analyzed circRNA transcript profiles of PBMCs from T1DM children (n = 4) and age-matched healthy controls (n = 4) by circRNA microarray. Using a two-fold change and *p* < 0.05 as the threshold to define up- or down-regulated circRNAs, we identified 27 upregulated and 31 downregulated differentially expressed circRNAs in T1DM patients compared with healthy controls (Figure 1A). We analyzed the composition of the differentially expressed circRNAs in light of the positions of circRNAs in the transcripts; the profile consisted of 27 exonic circRNAs (47%), three intronic circRNAs (5%), two exonic-intronic circRNAs (3%), 21 exonic-UTR circRNA (36%), and others (9%) (Figure 1B). Next, we evaluated the expression of 27 upregulated circRNAs in PBMCs from children with T1DM and healthy control subjects in an expanded cohort. The results showed that only *hsa_circ_0062444, hsa_circ_0009718* and* hsa_circ_0060450* were detectable and significantly upregulated in PBMCs from T1DM patients compared with healthy controls (Figure 1C). Furthermore, higher expression levels of *IL-6*, *IL-1β,* and *TNF-α* were also observed in T1DM patients (Figure 1D). Importantly, the positive correlation between *hsa_circ_0062444* and *IL-6, IL-1β* or* TNF-α* was validated in human patients with T1DM, while no correlations between the *hsa_circ_0009718* or* hsa_circ_0060450* and *IL-6, IL-1β* or* TNF-α* were identified (Figure 1E and Figure S2A). Furthermore, the ability of *hsa_circ_0062444* to differentiate T1DM patients from healthy subjects was assessed by receiver operating curve (ROC) analysis, which yielded an area under the curve of 0.839 (Figure 1F). Together, these findings implied that *hsa_circ_0062444* might have important role in T1DM pathogenesis.

Next, bioinformatics analysis showed that *hsa_circ_0062444* was 4291 nt long and was the back-spliced circular product of the last three exons of the *PPM1F* transcript; thus, as an exonic circRNA, it was named as *circPPM1F* (Figure 1G). To assess the role of *circPPM1F* in the regulation of subsets of PBMCs, we separated human PBMCs into T cells, B cells, and monocytes to detect the expression levels of *circPPM1F*. Surprisingly, *circPPM1F* was mainly expressed in monocytes rather than T and B cells (Figure S2B). Considering monocytes can transform into macrophages in inflammatory tissues and *circPPM1F* was closely associated with inflammatory cytokines in T1DM patients, we subsequently focused on the regulatory role of *circPPM1F* in LPS-induced M1 macrophage activation.

### *circPPM1F* promoted M1 macrophage activation through enhancement of NF-κB signaling

**Figure 2:**
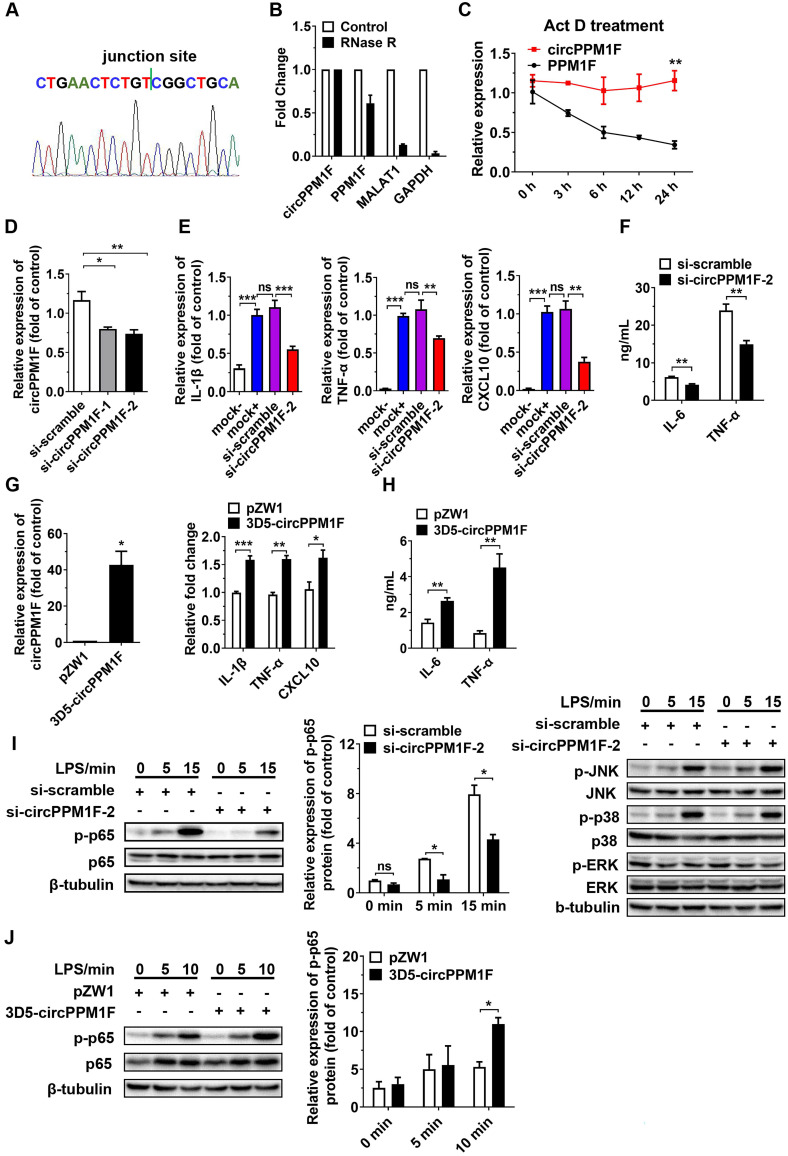
***circPPM1F* promotes M1 macrophage activation by enhancing NF-κB signaling. A.** The location of *circPPM1F* in the *PPM1F* transcript was validated by Sanger sequencing. **B.** Total RNA was digested with or without RNase R, followed by quantitative real-time PCR (qRT-PCR) measurements of *circPPM1F*, *PPM1F*, *MALAT1*, and *GAPDH*. **C.** The stability of *circPPM1F* was detected by qRT-PCR in THP1 macrophages after actinomycin D (Act D) treatment. **D.** qRT-PCR analysis of *circPPM1F* expression levels in THP1 macrophages following *circPPM1F* knockdown by two distinct siRNAs. **E.** qRT-PCR analyses of *IL‐1β, TNF‐α, and CXCL10* in THP1 macrophages with conditional treatment. Mock-, untransfected, and unstimulated cells; mock+, LPS stimulated alone cells; si-scramble, LPS-stimulated cells following transfection with si-scramble; si-circPPM1F-2, LPS-stimulated cells following transfection with si-circPPM1F-2. **F.** ELISA analyses of secreted cytokine levels in THP1 macrophages with *circPPM1F* knockdown, followed by LPS treatment. **G.** qRT-PCR analyses of *circPPM1F* in THP1 macrophages with 3D5-*circPPM1F* or pZW1 transfection (left); M1-associated gene expressions in LPS stimulated THP1 macrophages overexpressing *circPPM1F* were quantified by qRT-PCR analysis (right). **H.** ELISA analyses of secreted cytokine levels in *circPPM1F*-overexpressed THP1 macrophages, followed by LPS treatment. **I.** Western blot showing total p65, p38, ERK1/2, JNK and their phosphorylation levels in THP1 macrophages with or without *circPPM1F* knockdown after LPS treatment. **J.** Western blotting analysis to evaluate levels of total p65, phosphorylated p65 in *circPPM1F*-overexpressed THP1 macrophages. The levels of p-p65 were normalized to that of β-tubulin and quantified using Image J software. Data are presented as mean ± SEM from three independent experiments. *p ≤ 0.05, **p ≤ 0.01, ***p ≤ 0.001, ns indicates no significance.

To verify whether *circPPM1F* was a truly circRNA and not a linear RNA, we performed RT-PCR and Sanger sequencing assays. A strict concordance between the sequencing results and the public *circPPM1F* sequence in circBase (http://www.circbase.org/cgi-bin/simplesearch.cgi) was observed (Figure 2A). Furthermore, we found the endogenous expression of *circPPM1F* was resistant to excessive ribonuclease R (RNase R) digestion while linear mRNAs were severely degraded (Figure 2B). Additionally, the stability of *circPPM1F* after Act D treatment in THP1 macrophages was examined. We found that *circPPM1F* was highly stable, with a half-life > 24 h, whereas *PPM1F* mRNA was readily degraded and had a half-life < 6 h (Figure 2C). Collectively, this evidence suggested that endogenous *circPPM1F* was truly a circular RNA.

To determine the functional influence of *circPPM1F* on M1 macrophage activation, we first knocked down the expression of *circPPM1F* in THP1-derived macrophages with two small interfering RNAs (siRNAs) targeting the *circPPM1F* junction site; the knockdown efficiency was confirmed by qRT-PCR and si-circPPM1F-2 was selected for the later study (Figure 2D). We found the expression of M1 macrophage associated genes (*IL-1β, TNF-α* and* CXCL10*) was downregulated in the cells with *circPPM1F* knockdown after LPS stimulation (Figure 2E). Meanwhile, the IL-6 and TNF-α protein expression were suppressed in the supernatant (Figure 2F). Further, in the “gain-of-function” studies with ectopically expressing *circPPM1F*, we found the expression of *IL-1β, TNF-α,* and* CXCL10* were upregulated following LPS stimulation (Figure 2G), concomitant with significantly enhanced IL-6 and TNF-α protein levels in the supernatant (Figure 2H). Taken together, these findings suggested that *circPPM1F* can promote M1 macrophage activation.

Upon activation, LPS-TLR4 augments macrophage activity through the production of inflammatory cytokines, and activation of NF-κB and MAPK pathways 24. To explore signaling pathways involved in *circPPM1F* regulation of M1 macrophage activation, we investigated the effects of *circPPM1F* on NF-κB and MAPK pathways. Intriguingly, western blotting showed that *circPPM1F* knockdown did indeed result in markedly reduced levels of phosphorylated p65 in THP1 macrophages, whereas ectopic expression of *circPPM1F* significantly increased the levels of phosphorylated p65. However, THP1 macrophages with *circPPM1F* knockdown exhibited no differences in phosphorylation levels of JNK, p38, and ERK (Figure 2I-J). Overall, these findings implied that *circPPM1F* can promote M1 macrophage activation by enhancing NF-κB signaling.

### *circPPM1F* inhibited PPM1F translation through competitively binding to HuR

**Figure 3:**
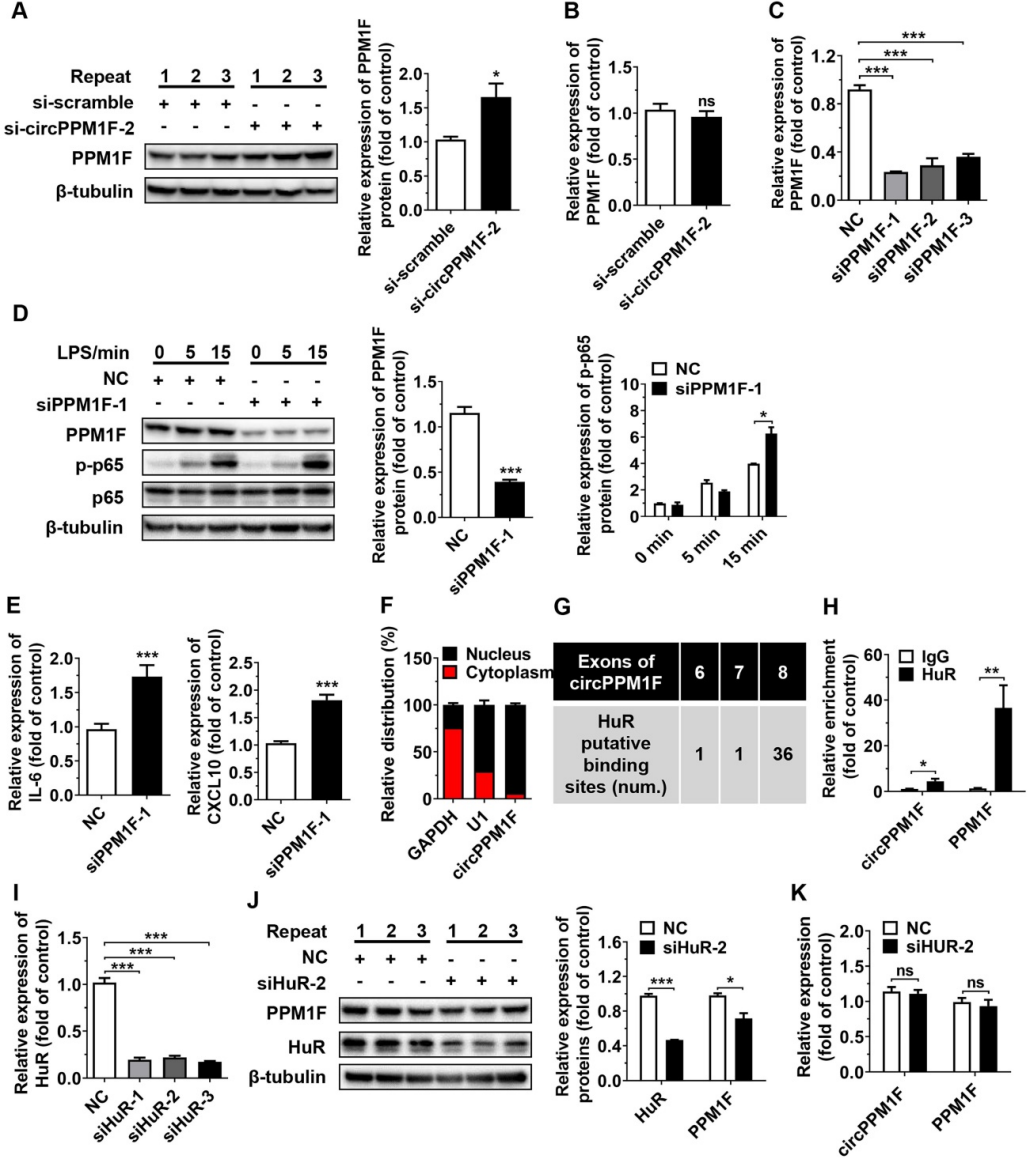
***circPPM1F* competitively binds to HuR to impair PPM1F translation. A.** Western blotting analysis to evaluate levels of PPM1F in *circPPM1F* knocked down-THP1 macrophages. **B.** qRT-PCR analyses of *PPM1F* expression in THP1 macrophages with *circPPM1F* knockdown. **C.** Analysis of *PPM1F* expression in THP1 macrophages transfected with *PPM1F* siRNA (200 nM) or control siRNA by qRT-PCR. **D.** Western blot analysis of PPM1F, p65, and p-p65 in THP1 macrophages with *PPM1F* knockdown, followed by LPS stimulation. **E.** Quantitative real-time PCR (qRT-PCR) analysis of M1-associated gene expression in THP1 macrophages with *PPM1F* knockdown, followed by LPS stimulation. **F.** qRT-PCR results showing the distribution of *circPPM1F* in the cytoplasmic and nuclear fractions of THP1 macrophages. *GAPDH* as cytoplasm control transcript, and *U1* as nuclear control transcript. **G.** Putative HuR binding sites within *circPPM1F* full-length sequence. **H.** The enrichment levels of *circPPM1F* and *PPM1F* in the products of the RNA immunoprecipitation (RIP) assay (HuR IP compared with IgG IP) as detected by qRT-PCR. **I.** qRT-PCR analysis of *HuR* in THP1 macrophages transfected with *HuR* siRNA (200 nM) or control siRNA. **J.** Protein levels of PPM1F and HuR were detected by western blotting in THP1 macrophages with HuR knockdown. **K.** qRT-PCR analysis of *circPPM1F* and *PPM1F* in THP1 macrophages with HuR knockdown. The levels of PPM1F, p-p65 and HuR were normalized to those of β-tubulin and quantified using Image J software. Data are presented as mean ± SEM from three independent experiments. *p ≤ 0.05, **p ≤ 0.01, ***p ≤ 0.001, ns indicates no significance.

PPM1F, a member of the PP2C family of Ser/Thr protein phosphatases, is a negative regulator of the IKK-NF-κB pathway through its effects on the dephosphorylation of TAK1 25. As *circPPM1F* is the back-spliced circular product of the coding gene, *PPM1F,* and circRNAs have been reported to exert regulatory effects on host genes at both transcriptional and post-transcriptional levels 26, 27, we questioned whether the effects of *circPPM1F* on NF-κB signaling activation were due to its influence on *PPM1F* expression. To verify our hypothesis, the levels of PPM1F protein and mRNA expression were determined in THP1 macrophages following *circPPM1F* knockdown. Surprisingly, knockdown of *circPPM1F* resulted in a significant increase of PPM1F protein expression, with no changes in its mRNA expression (Figure 3A-B), indicating that *circPPM1F* negatively regulated the expression of *PPM1F* at the translational stage. Furthermore, we knocked down the expression of PPM1F in macrophages and found phosphorylation of p65 and the expression of *IL-6*, and *CXCL10* were increased following LPS stimulation (Figure 3C-E). Taken together, these data implied that *circPPM1F* suppressed *PPM1F* translation, thereby facilitating NF-κB pathway and M1 macrophage activation.

Subcellular localization contributes to the modulatory mechanism of circRNAs on their targets. To further explore the mechanism underlying *circPPM1F*-mediated effects on *PPM1F*, we performed subcellular fractionation and localization assays. Surprisingly, in contrast to the cytoplasmic localization observed for a large number of verified exonic circRNAs, *circPPM1F* was primarily localized to the nucleus of THP1 macrophages (Figure 3F). circRNAs in the nucleus are reported to regulate gene translation via interaction with RNA-binding proteins (RBPs). We therefore searched for all putative RBPs binding to *circPPM1F* using the circRNA interactome database (https://circinteractome.nia.nih.gov/RNA_Binding_Protein/rna_binding_protein.html). Notably, the analysis revealed that the RBP containing the most potential binding sites with the *circPPM1F* was HuR (Figure 3G). HuR, a member of the ELAVL family of RBPs and selectively bind to AU-rich elements (AREs) within the 3′-untranslated regions (UTRs) of mRNA, stabilizing ARE-containing mRNA to regulate gene translation 28. To determine whether the interaction between *circPPM1F* and HuR was responsible for *circPPM1F*-mediated translational inhibition of *PPM1F*, we conducted RIP using an antibody specific for HuR and found a 5-fold enrichment of *circPPM1F* and 30-fold enrichment of *PPM1F* when the anti-HuR antibody was used, relative to use the IgG control (Figure 3H). These results thus implied a direct interaction of HuR with *circPPM1F* or *PPM1F.*

Next, we explored whether HuR is involved in the effects of *circPPM1F* on *PPM1F* translation; thus, siRNA directed at HuR was transfected to knock down HuR expression in THP1 macrophages. The knockdown of HuR resulted in a significant decrease in protein expression of PPM1F in THP1 macrophages (Figure 3I-J), whereas no changes in *circPPM1F* and *PPM1F* mRNA expression were detected (Figure 3K). Collectively, these studies strongly suggested that *circPPM1F* could suppress *PPM1F* translation through competitive interaction with HuR, which subsequently promotes activation of M1 macrophages.

### EIF4A3 and FUS coordinately regulated *circPPM1F* expression

**Figure 4:**
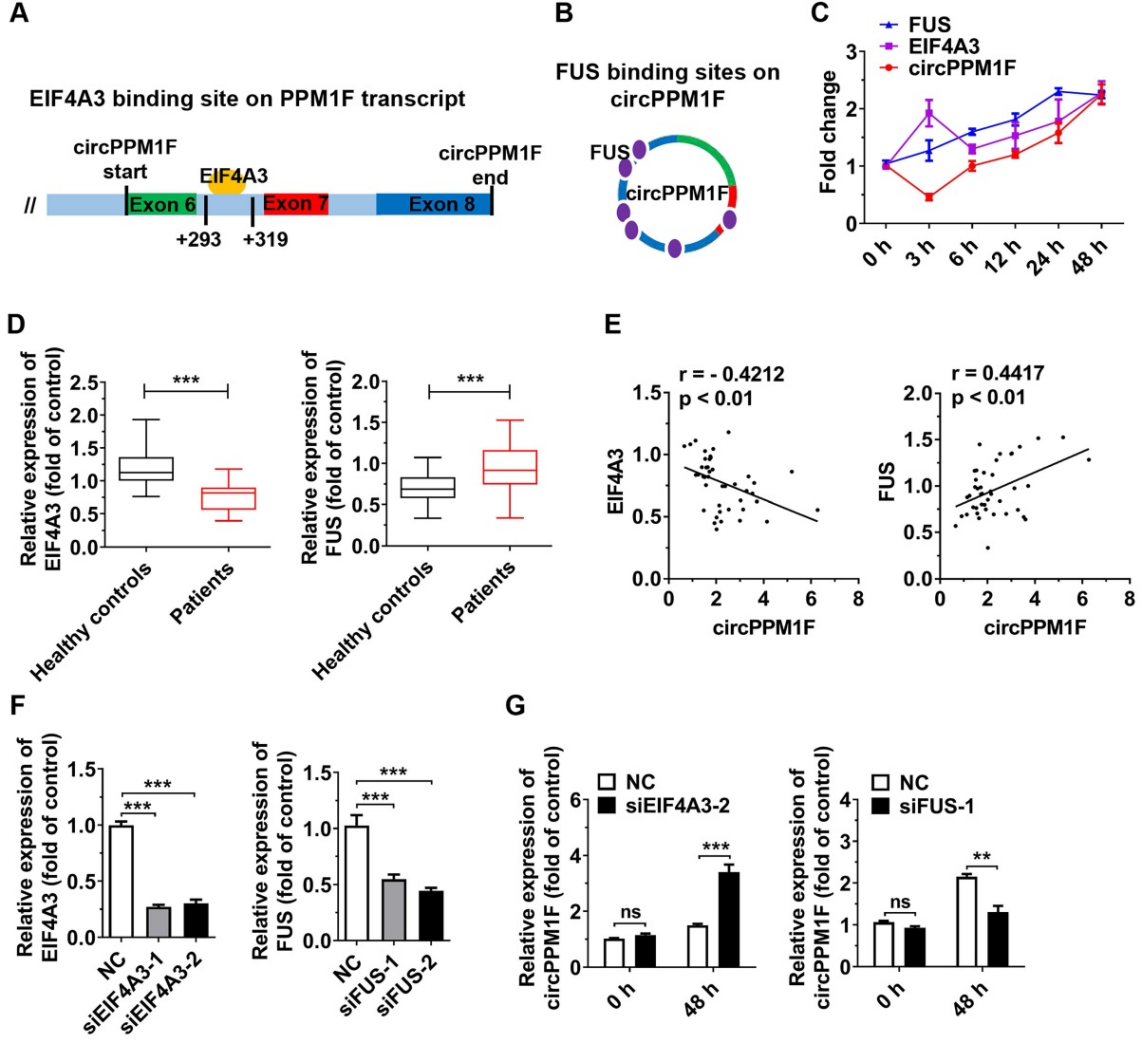
** EIF4A3 and FUS cooperatively regulate *circPPM1F* expression. A, B.** Bioinformatic prediction of binding sites of EIF4A3 (A) and FUS (B) on *circPPM1F*. **C.** Expression levels of *circPPM1F*, *EIF4A3*, and *FUS* were detected by quantitative real-time PCR (qRT-PCR) in THP1 macrophages treated with LPS. **D.** qRT-PCR analyses of the expression levels of *EIF4A3* and *FUS* in PBMCs from 43 T1DM patients and 45 healthy controls. **E.** Correlation analysis of the expression of *circPPM1F* and *EIF4A3*, or* FUS* levels in patients with type 1 diabetes mellitus (T1DM) (Pearson's correlation). **F.** qRT-PCR was used to measure the expression levels of *EIF4A3* and *FUS* in THP1 macrophages with *EIF4A3* or *FUS* knockdown. **G.** qRT-PCR analyses of the expression levels of *circPPM1F* in *EIF4A3* or *FUS* knocked down THP1 macrophages with or without lipopolysaccharide (LPS) treatment. Data are presented as mean ± SEM from three independent experiments. **p ≤ 0.01, ***p ≤ 0.001, ns indicates no significance.

Next, we explored the biogenesis of *circPPM1F*. Previous studies have shown that the biogenesis of circRNA depends on the spliceosomal machinery containing with cis- and trans-regulatory elements. Reverse intronic complementary sequences (ICS) induced “head-to-tail” splicing by bringing the 5′- and 3′-termini of an exon or of consecutive exons into spatial proximity 29, 30. However, there were no ICS in either of the intronic flanking regions of the splice junction sites of *circPPM1F*. Therefore, RBPs might be responsible for expression of *circPPM1F*. Using a bioinformatics method (https://circinteractome.nia.nih.gov/index.html), we queried all RBPs that were reported to be involved in circRNA generation. The result revealed that a binding site for EIF4A3 is present in the downstream region of the *circPPM1F* transcript, and FUS-binding sites were identified in the mature *circPPM1F* (Figure 4A-B). We then measured the levels of *circPPM1F*, *EIF4A3,* and *FUS* expression over time in THP1 macrophages stimulated by LPS. Interestingly, *FUS* exhibited a time-dependent increased expression following LPS treatment, while *EIF4A3* was upregulated at 3 h after stimulation, dropped to a basic level at 6 h, and thereafter was gradually upregulated. *circPPM1F* was downregulated at 3 h, but afterward its expression appeared to be gradually upregulated (Figure 4C). Furthermore, we analyzed the levels of *EIF4A3* and *FUS* expression in PBMCs from T1DM patients and healthy controls. The results indicated that the expression level of *EIF4A3* was significantly decreased, whereas that of *FUS* was increased in T1DM patients compared with healthy controls (Figure 4D). Importantly, there was a negative correlation between *EIF4A3* and *circPPM1F*, while a positive correlation was observed between *FUS* and *circPPM1F* (Figure 4E). The above results implied that *EIF4A3* and *FUS* may play important roles in *circPPM1F* expression.

To further investigate the roles of *EIF4A3* and *FUS* in *circPPM1F* expression, we knocked down *EIF4A3* and* FUS* expression in THP1 macrophages by transfection with their individual siRNAs (Figure 4F). Surprisingly, knockdown of *EIF4A3* or* FUS* resulted in no differences in the expression level of *circPPM1F* in THP1 macrophages without LPS treatment. However, knockdown of *EIF4A3* significantly elevated the expression level of *circPPM1F*, while knockdown of *FUS* decreased its level in THP1 macrophages treated with LPS (Figure 4G). Overall, these results implied that *EIF4A3* and *FUS* coordinately regulate *circPPM1F* expression during M1 macrophage activation.

### *circPPM1F* induced pancreatic β-cell apoptosis by promoting M1 macrophage activation* in vitro*

**Figure 5:**
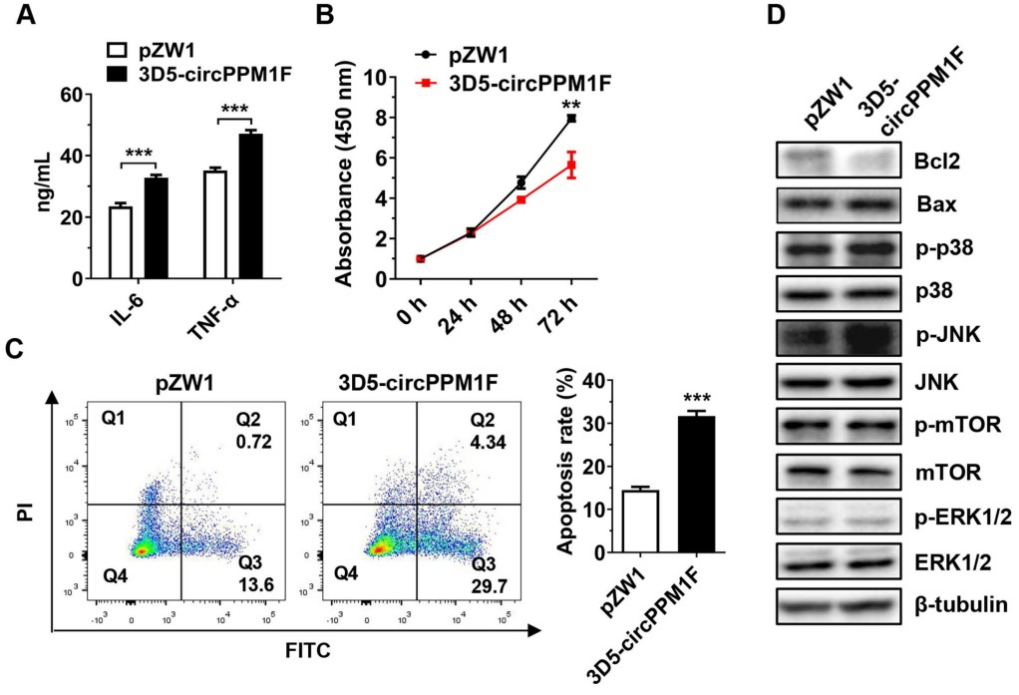
***circPPM1F* induces pancreatic β cell apoptosis through M1 macrophage activation. A.** ELISA analyses of secreted cytokine levels in conditional media from *circPPM1F*-overexpressed Raw264.7 cells, followed by LPS treatment. **B.** Following incubation with conditional media, cell proliferation of MIN6 cells was assessed by the CCK-8 assay. **C.** After the incubation with conditional media, MIN6 cells were stained with Annexin V-FITC and propidium iodide (PI) before fluorescence analysis by flow cytometry. The percentage of cells in the four different quadrants was calculated and the results presented in different histograms indicating the fraction of apoptotic cells were Annexin V^+^/PI^-^ and Annexin V^+^/PI^+^. **D.** Western blotting analysis to detect expression levels of Bcl2, Bax, p38, p-p38, JNK, p-JNK, ERK1/2, p-ERK1/2, mTOR, and p-mTOR in MIN6 cells cultured with conditional media. Data are presented as means ± SEM from three independent experiments. **p ≤ 0.01, ***p ≤ 0.001.

To assess the pathological effects of *circPPM1F*-induced M1 macrophage activation on pancreatic β-cells, we performed a cell co-culture assay with Raw264.7 cells overexpressing *circPPM1F* and murine pancreatic β-cells MIN6. Consistent with the mRNA levels, the IL-6 and TNF-α protein levels were enhanced in conditional media from Raw264.7 cells overexpressing *circPPM1F* (Figure 5A). Notably, we found that the proliferation rate of MIN6 cells was significantly inhibited by the conditional media (Figure 5B). In addition, the media markedly enhanced the rate of apoptosis in MIN6 cells (Figure 5C). Previous studies have indicated that mTOR and MAPK pathways are crucial for pancreatic β-cell apoptosis 31, 32. To investigate the potential molecular mechanisms inducing MIN6 cell apoptosis, we confirmed the effects of the conditional media on mTOR and MAPK pathways in MIN6 cells via western blotting. Decreased expression levels of Bcl2 and elevated Bax were observed in MIN6 cells cultured with media from *circPPM1F* overexpressing Raw264.7, and this was accompanied by increased levels of p38 and JNK phosphorylation (Figure 5D). However, there were no apparent changes in the phosphorylation levels of mTOR and ERK (Figure 5D). Collectively, these data suggested that *circPPM1F*-mediated M1 macrophage activation could induce pancreatic β cell apoptosis through the MAPK pathway *in vitro*.

### *circPPM1F* exacerbated pancreas injury in STZ-induced diabetic mice through M1 macrophage activation

**Figure 6:**
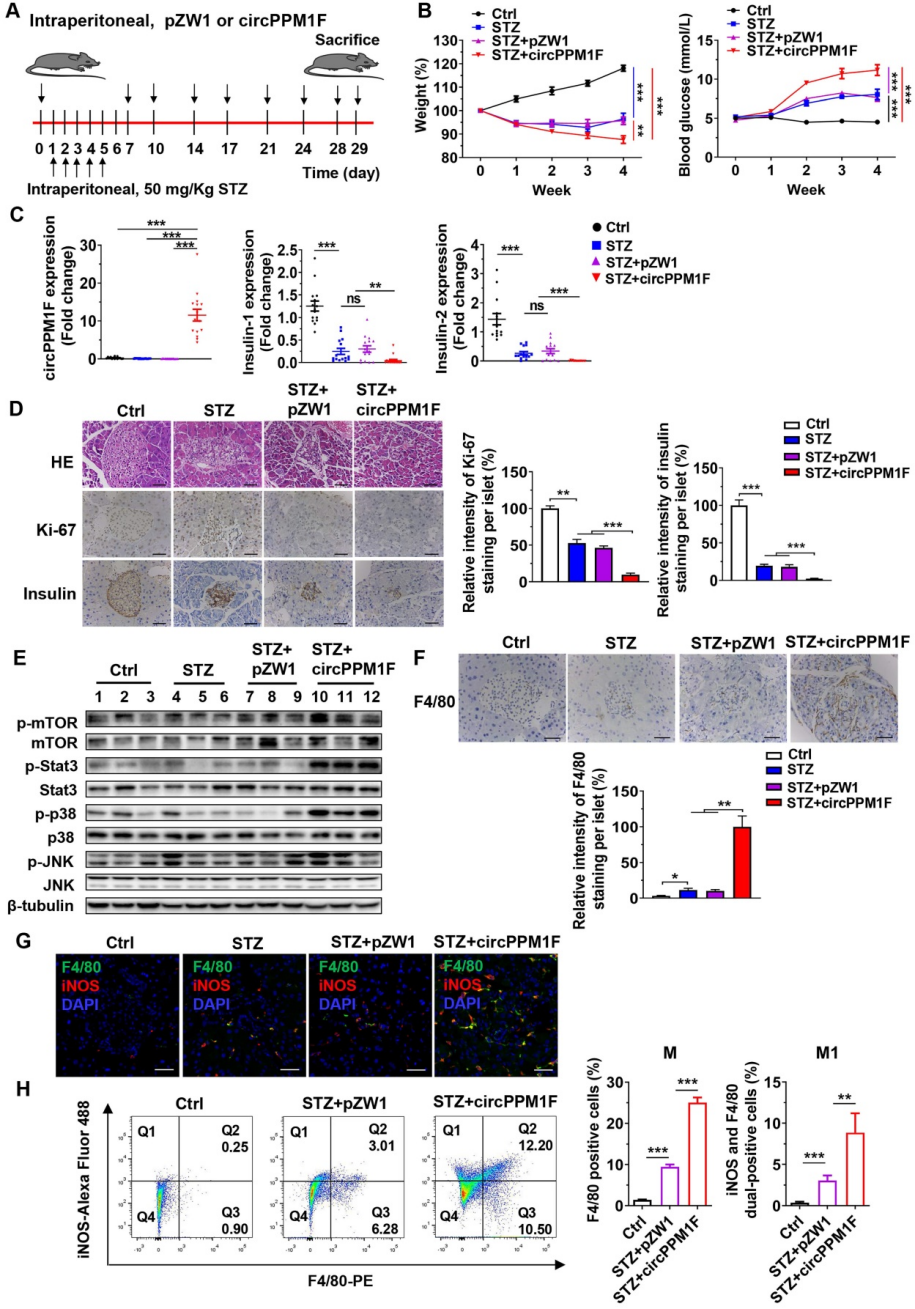
***circPPM1F* facilitates pancreatic islet injury in diabetic mice through M1 macrophage activation. A.** Treatment of circPPM1F in the STZ-induced diabetic mouse model (15 mice per group). **B.** Mean weekly body weight (left) and fasting blood glucose (right) change in four-group mice. **C.** Levels of *circPPM1F* and insulin in pancreas tissues were detected by quantitative real-time PCR (qRT-PCR). **D.** Representative hematoxylin and eosin (H&E)-stained pancreas tissues, immunohistochemistry (IHC) images of Ki-67 and insulin expression in pancreatic islets from experimental mice. Scale bar indicates 50 µm. Semi-quantification of Ki-67 and insulin staining of per islet were done by using Image J software. **E.** The levels of total p38, JNK, Stat3, mTOR, and their corresponding phosphorylated forms in pancreas tissues from experimental models were quantified by western blot. **F.** Representative IHC images of F4/80 expression in pancreatic islets from experimental mice. Scale bar indicates 50 µm. Semi-quantification of F4/80 staining of per islet were done by using Image J software. **G.** Immunofluorescence staining of infiltrated F4/80^+^/iNOS^+^ M1 macrophages in mice pancreatic islets. Green represents anti-F4/80 Ab; red represents anti-iNOS Ab; yellow represents F4/80 and iNOS merged; blue represents DAPI. Scale bar indicates 50 µm. **H.** The percentages of M1 macrophages in pancreas tissue cells from STZ-treated mice with or without *circPPM1F* overexpression and control mice were determined by flow cytometry (left). Quantification analyses of macrophages (F4/80^+^) and M1 macrophages (F4/80^+^/iNOS^+^) in pancreas tissue cells (right). *p ≤ 0.05, **p ≤ 0.01, ***p ≤ 0.001.

To assess the role of *circPPM1F* in pancreas injury of diabetic mice, we generated a diabetic mouse model using intraperitoneal (*i.p.*) injection of STZ (Figure 6A). We first investigated the dynamic expression profiles of *circPPM1F* in the pancreas, liver, spleen, and PBMCs of mice injected with *circPPM1F* or pZW1 plasmid for 3, 5, and 7 days. The results showed that the level of *circPPM1F* expression was highest in pancreas compared with that in the liver, spleen and PBMCs, and peaked on day 5 (Figure S3A). Next, successful induction of the diabetic mouse model was evidenced by significant weight loss one week after initial injection of STZ, and hyperglycemia two weeks after the injection. Importantly, the STZ+circPPM1F group mice achieved more severe weight reduction and hyperglycemia than STZ or STZ+pZW1 treated mice groups (Figure 6B). Further, the levels of *circPPM1F* and *insulin* expression in pancreas tissues were measured. Mice in the STZ+circPPM1F group displayed significantly enhanced *circPPM1F* and decreased *insulin* expression in the pancreas compared with the other three groups (Figure 6C). Furthermore, we assessed the effects of *circPPM1F* on pancreatic injury. The expression levels of inflammatory cytokines, oxidative stress indicators, indicators related to insulin secretion and cell apoptosis were detected. Specifically, STZ+circPPM1F group mice exhibited significantly higher levels of *IL-6, IL-1β, iNOS,* and* Bax* expression, accompanied by lower levels of *Sod, Cat, GSH-Px, Glut2, Gck,* and* Bcl2* expression, relative to control, STZ, and STZ+pZW1 groups (Figure S3B). Similar to the results in *Bcl2 and Bax* at the mRNA level, decreased levels of Bcl2 and enhanced Bax protein were detected in mice of the STZ+circPPM1F group compared with those of the STZ+pZW1 group (Figure S3C).

Pancreatic dysfunction usually represents pathological changes in the diabetic pancreas. Notably, Histological analysis showed that STZ and STZ+pZW1 groups displayed distinctly abnormal islets structure as compared with the control group, appearing as small islets, inhomogeneous islet cells, and cytoplasmic vacuolation. Specifically, STZ+circPPM1F group mice exerted more severe damage of islets. Furthermore, Ki-67 staining indicated decreased proliferation of pancreas cells in the STZ and STZ+pZW1 groups, and a greater decrease in the STZ+circPPM1F group, compared with the control group. In addition, similar to the mRNA expression patterns observed for insulin in the experimental mice, protein levels of insulin were significantly lower in mice overexpressing *circPPM1F* (Figure 6D). Altogether, these findings indicated that *circPPM1F* could aggravate the pancreas injury of STZ-induced diabetic mice.

Subsequently, we wanted to examine the molecular mechanism of pancreas injury. Considering that the Stat3, MAPK, and Akt-mTOR pathways play a prominent role in oxidative stress, inflammatory response, and cell apoptosis 33, 34, we measured the levels of Stat3, p38/JNK, mTOR and their corresponding phosphorylated protein expression. Importantly, the results showed that the mice of the STZ+circPPM1F group exhibited significantly enhanced levels of phosphorylated Stat3, p38, and JNK expression compared with control, STZ and STZ+pZW1 groups. However, there were no differences in the phosphorylation levels of mTOR (Figure 6E). Therefore, these data suggested that *circPPM1F* could increase pancreas injury by activating MAPK and Stat3 pathways *in vivo*.

In order to further evaluate effects of *circPPM1F* on macrophage activation in pancreas islets of diabetic mice, we assessed the infiltration of macrophages into islets cells. Compared with control mice, increased levels of F4/80^+^ cells were observed in pancreas islets in STZ and STZ+pZW1 group mice, and the highest levels of F4/80^+^ cells were detected in the STZ+circPPM1F group (Figure 6F). Moreover, to demonstrate whether *circPPM1F* promoted infiltration of macrophages into the islet cells due to M1 macrophage activation, we detected levels of F4/80^+^/iNOS^+^ cells by immunofluorescence staining. Notably, elevated levels of F4/80^+^/iNOS^+^ cells were observed in pancreas islets of mice from the STZ+circPPM1F group compared with that from mice with or without STZ treatment (Figure 6G). In addition, the levels of M1 macrophage activation in the pancreas were validated by flow cytometry. Similar to the positive patterns of F4/80 and iNOS observed in immunohistochemistry (IHC) and immunofluorescence assays, the STZ+circPPM1F group mice displayed higher frequencies of macrophages (F4/80^+^) and M1 macrophages (F4/80^+^/iNOS^+^) in comparison to the other groups (Figure 6H). Taken together, these findings implied that *circPPM1F* could facilitate injury of pancreatic islets in diabetic mice by promoting M1 macrophage activation.

## Discussion

In this study, we showed for the first time that *circPPM1F* was overexpressed in PBMCs from T1DM patients, and it increased M1 macrophage activation through the *circPPM1F*-HuR-PPM1F-NF-κB axis. Moreover, EIF4A3 and FUS were critical for coordinately controlling *circPPM1F* expression. In addition, *circPPM1F* could accelerate pancreatic islet cell apoptosis in diabetic mice by promoting the activation of M1 macrophages. Thus, we speculate that *circPPM1F* might potential represent a new therapeutic target for T1DM, adding a new dimension to the functional importance of circRNA regulation in diabetes mellitus.

Exonic circRNAs are usually abundant in the cytoplasm and function mainly through the microRNA “sponge” mechanism 35. In contrast, intron-containing circRNAs (intronic circRNAs and exon-intron circRNAs), in general, are enriched in the nucleus and are involved in regulation of host gene expression 36. However, Errichelli et al. found that three completely spliced exonic circRNAs were almost exclusively located to the nucleus 37. Consistent with this finding, in our study, as an exonic circRNA, *circPPM1F* was also constitutively expressed in the nucleus rather than in the cytoplasm of THP1 macrophages. Among the predicted RBPs of *circPPM1F,* we found 14 binding sites for fragile X mental retardation 1 (FMR1, also known as FMRP) were present in *circPPM1F*. Interestingly, FMRP participated in RNA trafficking from the nucleus to the cytoplasm, displaying different subcellular distribution due to alternative splicing 38, 39. We speculated that FMRP might be responsible for the nuclear and cytoplasmic localization of *circPPM1F*. It would be helpful to investigate the interaction of FMRP with *circPPM1F* to better understand the nuclear location of *circPPM1F*.

Recently, circRNAs have attracted increasing attention for their potential roles in regulating parental gene expression 26, 27. Meanwhile, parental genes may also be involved in circRNA biosynthesis 40. PPM1F has been reported to regulate cancer cell growth and metastasis 41, whereas its roles in the pathogenesis of T1DM and M1 macrophage activation remain unclear. Notably, our findings provide evidence supporting the hypothesis that circPPM1F-mediated M1 macrophage activation may be attributable to reduced protein levels of the *PPM1F* gene. Recently, HuR is a well-studied RBP that positively augments stability of a number of linear mRNAs and ncRNAs, but also binds to introns of pre-mRNAs to modulate splicing 28. It has been revealed that the interaction of HuR and circPABPN1 impaired the normal interaction of HuR's with linear mRNAs, especially parental pre-mRNA of circPABPN1, which consequently suppressed the production of *PABPN1* protein 42. Importantly, our data provide further evidence that HuR is a key contributor to *circPPM1F* and *PPM1F* interactions. Instead of impacting on *circPPM1F* transcription, the interaction with HuR and *circPPM1F* prevented HuR binding to *PPM1F* mRNA, resulting in a reduction of *PPM1F* translation. Such a relationship between circRNA and host mRNA is conceptually intriguing, and it might be generalizable to other RBPs. Additionally, our study was the first to reveal the role of PPM1F in M1 macrophage activation, implying that the *circPPM1F*-HuR-PPM1F axis may represent a novel potential therapeutic target in T1DM.

RBPs are required for regulation of the biogenesis specific circRNAs in a positive or negative way. In this study, our data showed that EIF4A3 and FUS oppositely regulated *circPPM1F* expression in response to external stimuli. EIF4A3, a member of the DEAD box protein family, has been implicated in nuclear and mitochondrial splicing, ribosome and spliceosome assembly, and translation initiation. Recent studies have revealed that EIF4A3 promoted circMMP9 and circSEPT9 expression via binding to parental pre-mRNAs 43, 44. However, in our study we found that EIF4A3 could inhibit *circPPM1F* expression. Unexpectedly, we identified a binding site for EIF4A3 in the downstream region (i.e., at intron 6 of *PPM1F* transcript) of *circPPM1F*. Therefore, we assume that this unusual binding site might be responsible for the suppression of EIF4A3 on *circPPM1F* biogenesis. In addition, Errichelli et al. found FUS either increased or repressed circRNA biogenesis by binding to the introns flanking the back-splicing junctions, and the interaction machinery could control a complex interplay between linear and back-splicing 37. In contrast to back-splicing regulation, our data indicated that FUS might also bind to mature *circPPM1F* at the post-transcriptional level and positively mediate its expression upon stimulation. Overall, these findings demonstrated that diverse RBPs participate in circRNA biogenesis, and that some may contribute to diverse regulatory mechanisms, even antagonistically. Hopefully, our study will prompt future work exploring RBP-based diverse regulation of circRNA biogenesis.

STZ is a broad-spectrum antibiotic possessing antitumor, oncogenic, and diabetogenic properties, and multiple small dose injections of STZ in mice produce pancreatic insulitis, with progression to nearly complete β-cell destruction and diabetes mellitus 45. Although the timing and appearance of the inflammatory islet lesions do not demonstrate that STZ stimulation acts by initiating a cell-mediated immune reaction, multiple low doses of STZ have been shown to selectively destruct β-cells, which in turn induces immune reactions against pancreatic islets, leading to β-cell apoptosis and subsequently diabetes mellitus. This model resembles the key features of T1DM patients with a loss of β-cell function and the development of hyperglycemia 46-48. At present, the STZ-induced mouse model is one of the most widely used animal models of human autoimmune diabetes in T1DM studies 46, 49-51. In our study, we focused the effect of *circPPM1F* on development of STZ-induced diabetes mellitus. Consistent with *in vitro* studies of increased pancreatic β-cell apoptosis, we found that *circPPM1F*-mediated M1 macrophage activation could also facilitate pancreas injury in diabetic mice. However, it remains to be clarified whether macrophage-specific expression of *circPPM1F* facilitates the development of T1DM. Treatment of *circPPM1F* with a macrophage-specific promoter in the STZ-treated NOD mouse model, or generating a chimeric, macrophage-specific *circPPM1F* knocked-in T1DM mouse model would be helpful to address the issue.

In summary, our studies demonstrate the positive role of *circPPM1F* in LPS-induced M1 macrophage activation through the *circPPM1F*-HuR-PPM1F-NF-κB axis. *In vivo*, *circPPM1F* facilitated pancreas injury in STZ-induced diabetic mice by promoting M1 macrophage activation. Additionally, EIF4A3 and FUS might be required for the maintenance of *circPPM1F* expression during the progression of T1DM. Taken together, our work provides new insights into the pathogenesis of T1DM and suggests a potential novel biomarker or therapeutic target for T1DM.

## Supplementary Material

Supplementary figures and tables.

## Abbreviations

Act D: actinomycin D
circRNAs: circular RNAs
EIF4A3: eukaryotic initiation factor 4A-III
FMR1: fragile X mental retardation 1 (also known as FMRP)
FUS: fused in sarcoma
GEO: Gene Expression Omnibus
HuR: human antigen R
MAPK: mitogen-activated protein kinase
NF-κB: nuclear factor kappa B
mTOR: mammalian target of rapamycin
PPM1F: protein phosphatase, Mg^2+^/Mn^2+^ dependent 1F
PBMCs: peripheral blood mononuclear cells
qRT-PCR: quantitative real-time PCR
RBP: RNA-binding protein
RIP: RNA immunoprecipitation
ROC: receiver operating curve
siRNA: small interfering RNA
Stat3: signal transducers and activators of transcription 3
STZ: streptozocin
T1DM: type 1 diabetes mellitus
