# Supplementary material for: Circular RNA circPPM1F modulates M1 macrophage activation and pancreatic islet inflammation in type 1 diabetes mellitus
Source: Theranostics. 2020 Aug 29;10(24):10908–24. doi: 10.7150/thno.48264 (PMC7532688; doi:10.7150/thno.48264)
Supplement: Supplementary file 1 — Supplementary figures and tables. [file thnov10p10908s1.pdf]

Supplementary materials

Circular RNA *circPPM1F* modulates M1 macrophage activation and pancreatic islet inflammation in type 1 diabetes mellitus

Caiyan Zhang[1,2#], Xiao Han[1,2#], Lan Yang[1,2#], Jinrong Fu[1#], Chengjun Sun[3], Saihua Huang[1,2], Wenfeng Xiao[1,2], Yajing Gao[1,2], Qiuyan Liang[1,2], Xiang Wang[1,2], Feihong Luo[3], Wei Lu[3], Yufeng Zhou[1,2]*

[1] Institute of Pediatrics, Children's Hospital of Fudan University, and the Shanghai Key Laboratory of Medical Epigenetics, International Co-laboratory of Medical Epigenetics and Metabolism, Ministry of Science and Technology, Institutes of Biomedical Sciences, Fudan University, Shanghai 200032, China

[2] National Health Commission (NHC) Key Laboratory of Neonatal Diseases, Fudan University, Shanghai, China

[3]Department of Pediatric Endocrinology and Inherited Metabolic Diseases, Children's Hospital of Fudan University, Shanghai, 201102, China

[#]These authors have contributed equally to this work.

*To whom correspondence should be addressed:

Yufeng Zhou, M.D., Ph.D., 399 Wanyuan Rd, Minhang, Shanghai 201102, China

Telephone: 86-021-64932907. E-mail: yfzhou1@fudan.edu.cn

Figure S1

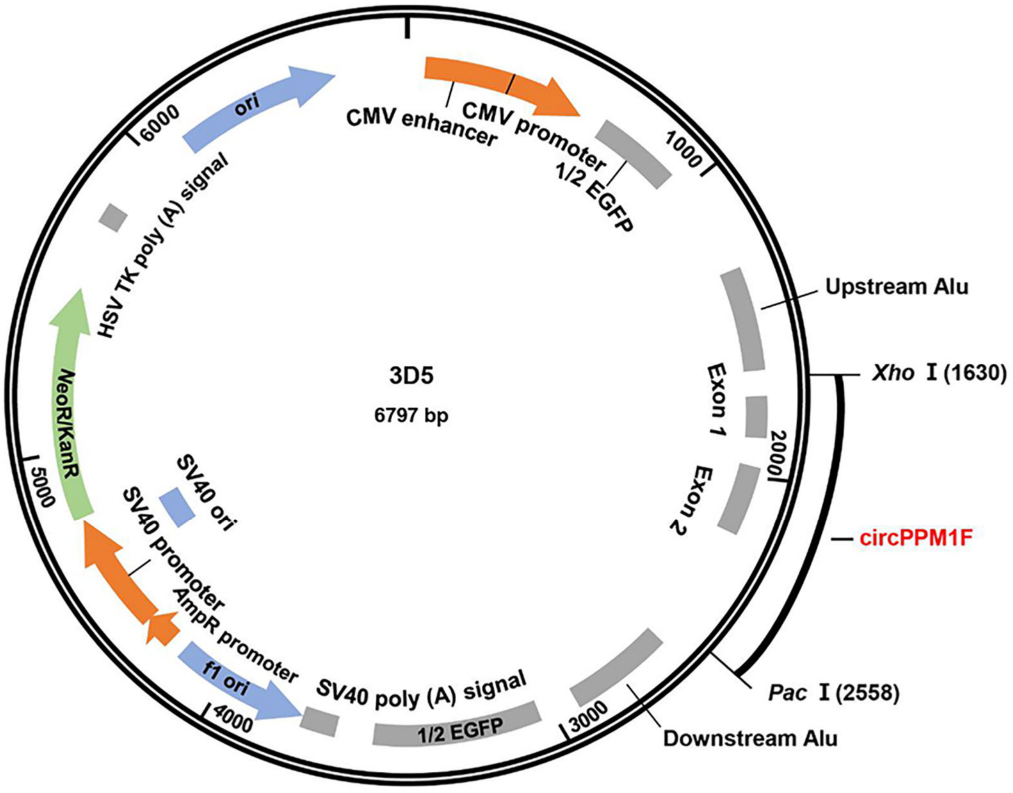

Figure S1. Schematic map of the *3D5-circPPM1F* vector.

Figure S2

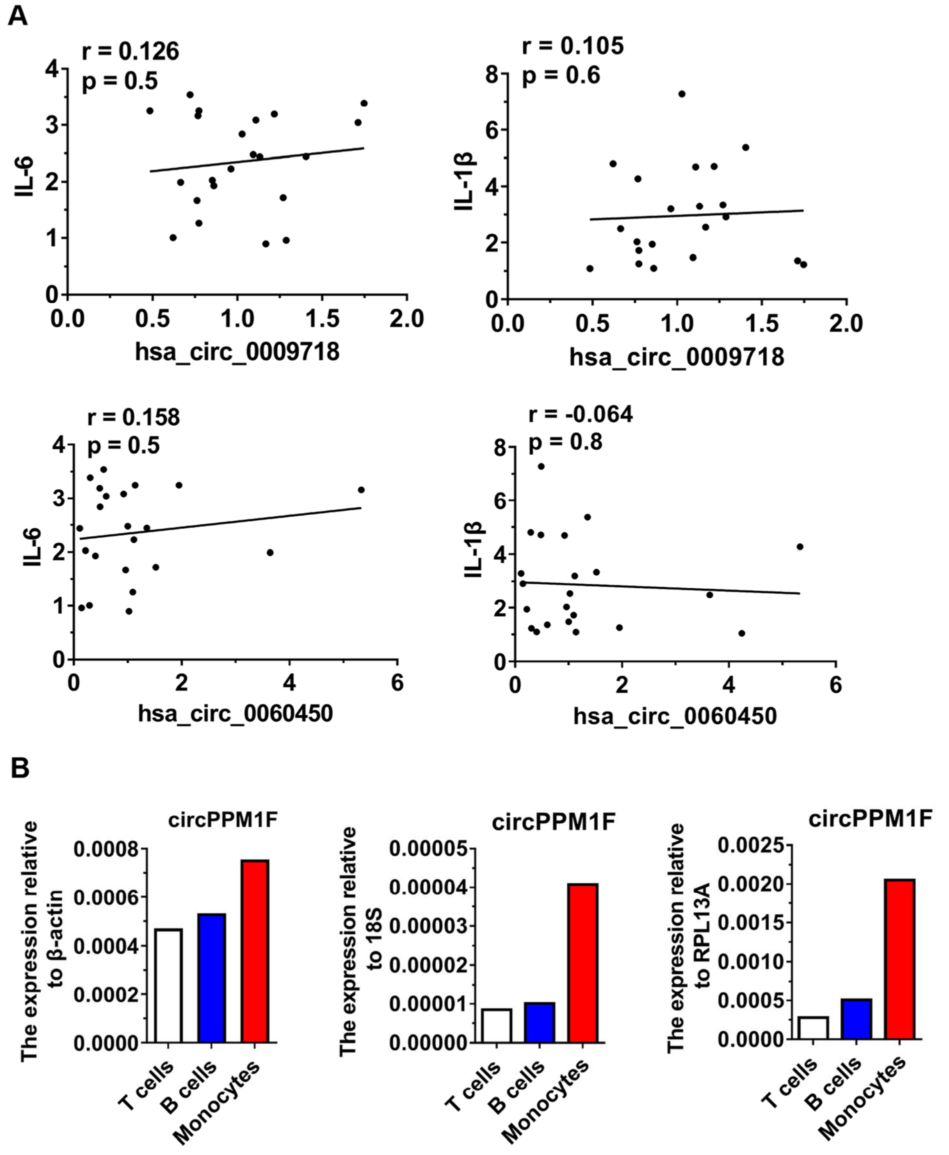

Figure S2. Expression of circRNAs in peripheral blood mononuclear cells (PBMCs). A. Correlation analysis of the expression of *hsa_circ_0009718* or *hsa_circ_0060450* and *IL-6*, or *IL-1β* levels in PBMCs from patients with type 1 diabetes mellitus (T1DM) (n = 22, Pearson's correlation). **B.** qRT-PCR assay was used to measure *circPPM1F* expression in monocytes, T cells, and B cells in PBMCs.

Figure S3

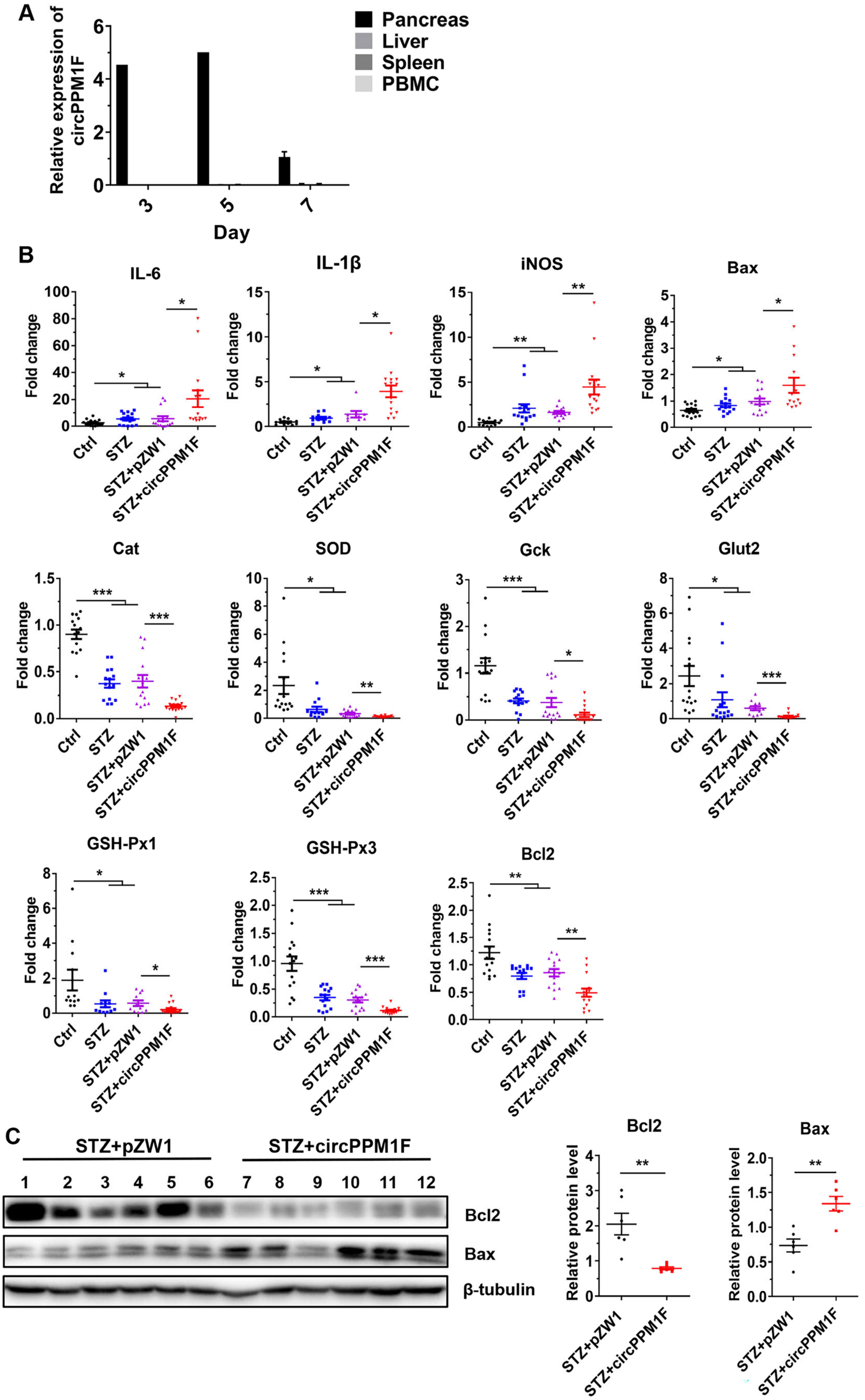

A
Relative expression of circPPM1F
Pancreas
Liver
Spleen
PBMC
Day
3
5
7
B
IL-6
IL-1β
iNOS
Bax
Cat
SOD
Gck
Glut2
GSH-Px1
GSH-Px3
Bcl2
Fold change
Ctrl
STZ
STZ+pZW1
STZ+circPPM1F
C
STZ+pZW1
STZ+circPPM1F
1
2
3
4
5
6
7
8
9
10
11
12
Bcl2
Bax
β-tubulin
Relative protein level

Figure S3. The evaluation of pancreatic injury in STZ-induced diabetic mouse model. A. Dynamic expression profiles of *circPPM1F* in pancreas, liver, spleen, and PBMCs from mice with injection of *circPPM1F* plasmid for 3, 5, or 7 days. **B.** qRT-PCR analyses of *IL-6, IL-1β, iNOS, Bax, Sod2, Cat, GSH-Px-1, GSH-Px-3, Glut2, Gck,* and *Bcl2* expression in pancreas tissues from experimental model mice. **C.** The levels of Bcl2 and Bax protein in 6 random pancreas tissue samples from STZ-treated mice with *circPPM1F* or pZW1 injection (left). The levels of Bcl2 and Bax were normalized to those of β-tubulin and quantified using Image J software (right). Data are presented as means ± SEM from three independent experiments. $^*p \leq 0.05$, $^{**}p \leq 0.01$, $^{***}p \leq 0.001$.

Table S1 Clinical characteristics of T1DM patients and healthy controls

Characteristic	Patients (n = 47)	Healthy controls (n = 49)
Sex, no. (M/F)	22/25	21/28
Age (y)	8.5 ± 0.6	8.0 ± 1.2
Duration (y)	1.8 ± 0.4	0
BMI (Kg/m^2)	16.7 ± 0.6	N/A
cGLU (mmoL/L)	18.0 ± 1.7	N/A
HbA1C (%)	11.0 ± 0.5 (ref. 4 ~ 6)	N/A
c peptide (ng/mL)	0.5 ± 0.1 (ref. 1.1 ~ 4.4)	N/A
GADA, no. (+/-)	8/39	0/49
IA-2A, no. (+/-)	21/26	0/49
IAA, no. (+/-)	7/40	0/49
ICA, no. (+/-)	9/38	0/49
Urine ketone, no. (+/-)	14/33	0/49

Data for age, BMI, cGLU, HbA1C and c peptide are expressed as means ± SEM.

Table S2 Primer sequences used for amplification

Name	Usage	Sequence (5′- 3′)
β-actin	qPCR forward	CACCATTGGCAATGAGCGGTTC
	qPCR reverse	AGGTCTTTGCGGATGTCCACGT
GAPDH	qPCR forward	GTCTCCTCTGACTTCAACAGCG
	qPCR reverse	ACCACCCTGTTGCTGTAGCCAA
U1	qPCR forward	GGGAGATACCATGATCACGAAGGT
	qPCR reverse	CCACAAATTATGCAGTCGAGTTTCCC
MALAT1	qPCR forward	CTTAAGCGCAGCGCCATTTT
	qPCR reverse	CCTCCAAACCCCAAGACCAA
circPPM1F	qPCR forward	TTGTGTTCCTGTGCAAGGGG
	qPCR reverse	TGCAGCCGACAGAGTTCAGA
hsa_circ_0009718	qPCR forward	TCACACCTAGCGAGAGCAGT
	qPCR reverse	TGCATACCTTGGTAGCCAGC
hsa_circ_0060450	qPCR forward	GGGAAGCAGTGCCGTGAAC
	qPCR reverse	GTCCTGCTGTCCAAACTGCCT
PPM1F	qPCR forward	GCTGCTACAGACAGACCTTTCC
	qPCR reverse	GGCGGTTAAAGAAACTCTGTGCC
HuR	qPCR forward	TGTTCTCTCGGTTTGGGCGGAT
	qPCR reverse	TCTTCTGCCTCCGACCGTTTGT
TNF-α	qPCR forward	CTCTTCTGCCTGCTGCACTTTG
	qPCR reverse	ATGGGCTACAGGCTTGTCACTC
IL-6	qPCR forward	AGACAGCCACTCACCTCTTCAG
	qPCR reverse	TTCTGCCAGTGCCTCTTTGCTG
IL-1β	qPCR forward	CCACAGACCTTCCAGGAGAATG
	qPCR reverse	GTGCAGTTCAGTGATCGTACAGG
CXCL10	qPCR forward	GGTGAGAAGAGATGTCTGAATCC
	qPCR reverse	GTCCATCCTTGGAAGCACTGCA
EIF4A3	qPCR forward	GGCACAGGAAAAACAGCCACCT
	qPCR reverse	TGTAGTCACCGAGAGCAAGCAG
FUS	qPCR forward	CAGACAGGGAAACTGGCAAGCT
	qPCR reverse	GGCGAGTAGCAAATGAGACCTTG
Primer1	Forward	TGAGATTTCATCAGGCTCGAGGTACGCCGCTGTCCACGT
	Reverse	TAGGGCTTCTGGAAGACATCCCCGATGGCTCTGGAG
Primer2	Forward	CTCCAGAGCCATCGGGGATGTCTTCCAGAAGCCCTA
	Reverse	GGCCAAAAACTTTTAATTAATCCTGTCACCTTCCTGCTG

Table S3 Sequences of NC and siRNAs

Name	Target mRNA	Sense sequence (5′- 3′)
si-circPPM1F (-1)	*circPPM1F*	CUGAACUCUGUCGGCUGCATT
si-circPPM1F (-2)	*circPPM1F*	CUCUGUCGGCUGCAGAGCGTT
si-PPM1F (-1)	PPM1F	GACAGACCUUUCCGAAUUCTT
si-PPM1F (-2)	PPM1F	AGCCUGGCACAGAGUUUCUTT
si-PPM1F (-3)	PPM1F	ACCGACCAGAUGUUUCUCATT
si-HuR (-1)	HuR	GAACGAAUUUGAUCGUCAATT
si-HuR (-2)	HuR	GCAGAUGUUUGGGCCGUUUTT
si-HuR (-3)	HuR	GGCUUUGUGACCAUGACAATT
si-EIF4A3 (-1)	EIF4A3	GCAUCUUGGUGAAACGUGATT
si-EIF4A3 (-2)	EIF4A3	CCGAGUGCUUAUUUCUACATT
si-EIF4A3 (-3)	EIF4A3	CGAUGAACGUUGCUGAUCUTT
si-FUS (-1)	FUS	CAGAGUUACAGUGGUUAUATT
si-FUS (-2)	FUS	GCCAAGAUCAAUCCUCCAUTT
si-FUS (-3)	FUS	GUGAGAAUGUUACAAUUGATT
NC	- -	UUCUCCGAACGUGUCACGUTT

Table S4 The details of all antibodies

Description (Anti-)	Sources	Cat./clone	Dilutions /WB	Dilutions /IHC/IF	Flow cytometry
PPM1F	Abcam, Cambridge, UK	ab200394	1 : 1000		
p65	CST, USA	#4764; C22B4	1 : 1000		
p-p65 (Ser536)	CST, USA	3033S; 93H1	1 : 1000		
p38	CST, USA	9212S	1 : 1000		
p-p38 (T180/Y182)	CST, USA	9216S; 28B10	1 : 1000		
JNK	CST, USA	9258S; 56G8	1 : 1000		
p-JNK (T183/Y185)	CST, USA	9251S	1 : 1000		
ERK1/2	CST, USA	4695S;137F5	1 : 1000		
p-ERK1/2 (T202/Y204)	CST, USA	4376S; 20G11	1 : 1000		
mTOR	CST, USA	2983S;7C16	1 : 1000		
p-mTOR (Ser2448)	CST, USA	5536S; D9C2	1 : 1000		
Stat3	CST, USA	9139S; 124H6	1 : 1000		
p-Stat3 (Y705)	CST, USA	9131S	1 : 1000		
HuR	CST, USA	#12582; D9W7E	1 : 1000		
Bcl2	CST, USA	15071S; 124	1 : 1000		
Bax, NT	EMD Millipore, USA	#06-499	1 : 1000		
β-Tubulin	Abcam, Cambridge, UK	ab6046	1 : 5000		
Ki-67	Signalway Ab, Texas, USA	#48871; 9N16		1 : 500	
Insulin	Proteintech, Chicago, USA	66198-1-Ig		1 : 1000	
F4/80 for IHC	CST, USA	#70076S		1 : 500	
F4/80 for IF	Servicebio	GB11027		1:8000	
F4/80	eBioscience	12-4801-80; BM8,			0.25 μg/T

iNOS	eBioscience	53-5920-80; CXNFT		0.06 μg/T
iNOS for IF	Servicebio	GB11119	1:500	
